# Developing the EPI Symptom Questionnaire (EPI-SQ): a qualitative study to understand the symptom experience of patients with exocrine pancreatic insufficiency (EPI)

**DOI:** 10.1186/s41687-024-00760-6

**Published:** 2024-07-25

**Authors:** Sally Mannix, Amit Bodhani, Leah Kleinman, Nikhil Khandelwal, Vikesh K. Singh

**Affiliations:** 1 Evidera Inc., Bethesda, MD USA; 2 AbbVie Inc., North Chicago, IL USA; 3 Evidera Inc., Seattle, WA USA; 4 Division of Gastroenterology, Johns Hopkins University School of Medicine, Baltimore, MD USA; 5 Digital Health, Oncology Research and Development, 1 Medimmune Way, Gaithersburg, MD 20878 USA

**Keywords:** EPI Symptom Questionnaire, Exocrine pancreatic insufficiency, Pancreatic exocrine insufficiency, Patient-reported outcome, Qualitative study

## Abstract

**Background:**

Symptom assessment is the key factor in determining disease status and optimal management of exocrine pancreatic insufficiency (EPI). There is a need for a standardized patient-reported outcome (PRO) questionnaire to assess symptoms in patients diagnosed with EPI. The purpose of this qualitative study was to increase understanding of the EPI symptom experience from the patients’ perspective, and to develop and evaluate the content validity of the EPI Symptom Questionnaire (EPI-SQ) in US patients with EPI.

**Methods:**

Concept elicitation interviews (Phase I) were conducted to understand the symptom experience in patients with a clinical diagnosis of EPI (i.e., fecal pancreatic elastase value of ≤ 200 mcg/g based on most recent value) due to chronic pancreatitis or pancreatectomy. The EPI-SQ was developed based on the data extracted from Phase I interviews and feedback from clinical experts. Next, separate cognitive interviews (Phase II) were conducted to evaluate participants’ understanding of the instructions, items, response scales, and recall periods of the instrument.

**Results:**

During Phase I interviews (*n* = 21), 19 participants (90%) reported abdominal pain as the most frequent EPI symptom and lifestyle changes were the most frequently endorsed impacts (*n* = 18; 86%). Phase II results indicated that all participants (*n* = 7) felt the 12-item EPI-SQ was relevant to their symptom experience and that they understood the items, instructions, and response options as intended.

**Conclusion:**

The qualitative data from this study support the content validity of the EPI-SQ in measuring EPI symptom severity in US patient populations diagnosed with EPI.

**Supplementary Information:**

The online version contains supplementary material available at 10.1186/s41687-024-00760-6.

## Background

Exocrine pancreatic insufficiency (EPI) is characterized by insufficient secretion or activity of exocrine pancreatic enzymes, resulting in maldigestion and malabsorption [1]. EPI is commonly associated with pancreatic disorders, such as chronic pancreatitis (CP), cystic fibrosis (CF), acute necrotizing pancreatitis, and pancreatic cancer. It can also occur because of pancreatic or gastrointestinal surgery [2, 3]. Due to its heterogenous nature, prevalence of EPI varies with severity of predisposing conditions:30–90% for CP, 80–90% for CF, 15–40% for acute pancreatitis, and 20–60% for unresectable pancreatic cancer. The prevalence of EPI after pancreatic surgery, depending on the type of resection, is estimated to be between 19% and 100% [1, 2, 4].

In most of the cases, EPI is undiagnosed or under-treated due to high variability in symptoms and their severity among patients or due to limitations of diagnostic tests [1, 3–5]. Delayed EPI diagnosis and treatment in affected patients potentially leads to worsening of symptoms and significant long-term consequences, including cardiovascular disease, osteoporosis, sarcopenia, and mortality due to EPI-associated malnutrition [2, 6, 7]. Given that EPI symptoms and severity vary between individuals, symptom assessment plays a crucial role in understanding disease status and optimal disease management in routine clinical practice.

Use of validated patient-reported outcome (PRO) instruments in capturing symptom experience from patients’ perspectives is well established [8–10]. PRO instruments would be useful tools in assessing EPI symptom severity, as well as in managing them appropriately [6]. Most PRO instruments currently used to monitor EPI symptoms are generic (e.g., 36-item Short Form of the Medical Outcomes Study [SF-36]) [11] or specific to other gastrointestinal conditions (e.g., European Organisation for Research and Treatment of Cancer Quality of Life Questionnaire C-30 [EORTC QLQ-C30], EORTC pancreatic cancer modules [PAN26 and PAN28], Gastrointestinal Quality of Life Index [GIQLI]) [12, 13].

To the research team’s knowledge, one disease-specific PRO instrument has been developed; the 18-item pancreatic exocrine insufficiency (PEI) Questionnaire (PEI-Q) that is designed to assess both EPI symptoms (abdominal symptoms and bowel movements) and impacts [3, 6]. The PEI-Q was developed in a European population (France, Germany, Spain, and the United Kingdom). Psychometric validation of the PEI-Q, using data from an observational trial in patients with CP or CF diagnosed with EPI, indicated that the instrument has good internal consistency, validity, and reliability, and discriminates according to EPI symptom severity [3]. However, half of the patients enrolled in the study were considered to have EPI based on expert clinician diagnosis rather than a diagnostic test [3].

At the time of initiation of the research presented in this publication, health care practitioners had no standardized means of monitoring symptoms in patients with EPI in the real-world setting. During the course of this research project, the PEI-Q was developed presenting an option for measurement of disease-related EPI symptoms and impacts; however, the availability of disease-specific PRO measures targeting assessment of EPI symptoms has remained very limited. Additional standardized, EPI-specific PRO questionnaires that specifically monitor symptoms in patients diagnosed with EPI represent a valuable contribution to EPI research and clinical management. The primary objective of Phase I of this qualitative study was to gain a broad understanding of living with the disease from the perspectives of patients diagnosed with EPI, with a specific focus on understanding patients’ perspectives on EPI symptoms. The primary objective of Phase II was to develop and evaluate the content validity of the EPI Symptom Questionnaire (EPI-SQ) in US patients diagnosed with EPI.

## Methods

### Study design

**Fig. 1:**
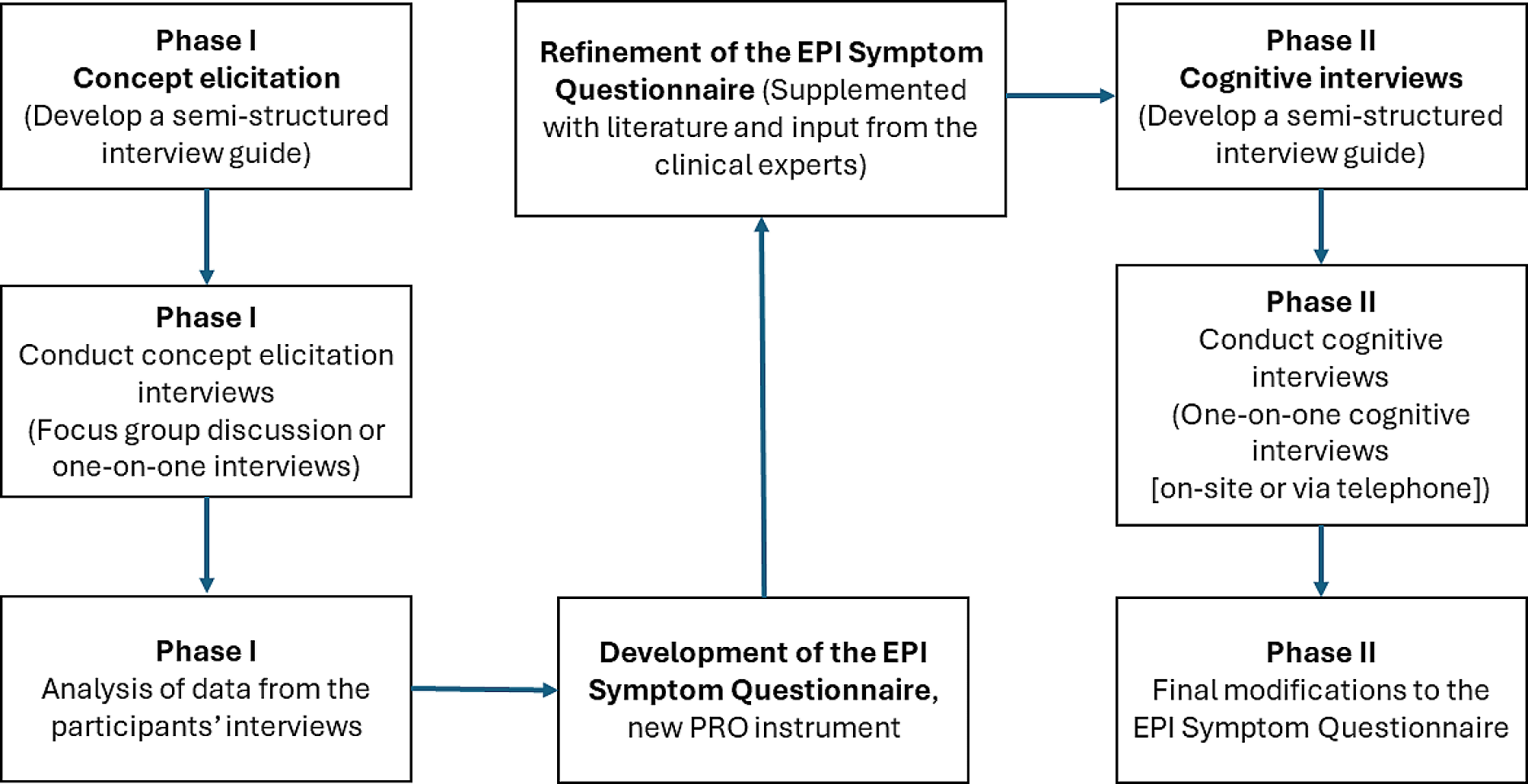
Overview of study design. *EPI* exocrine pancreatic insufficiency, *PRO* patient-reported outcome

This was a cross-sectional, qualitative study among patients diagnosed with EPI who were current or past users of pancreatic-enzyme replacement therapy (PERT) at the time of enrollment (Fig. 1). Semi-structured concept elicitation interviews (Phase I) and cognitive interviews (Phase II) were conducted following best practices outlined for developing and reporting on content validity of a new PRO instrument [8–10].

Concept elicitation interviews (Phase I) were used to identify key symptom concepts to be considered for inclusion in the new PRO instrument, which was developed based on Phase I results, literature, and input from clinical experts. The feedback of four clinical experts with experience in treating EPI was used to further refine the new PRO instrument. All four experts had at least seven years of experience in EPI diagnosis and treatment and rated each item (i.e., symptom) on relevance and likelihood to capture change over time. Finally, they provided any general feedback or comments for each item on the EPI-SQ. All responses from the clinical experts were consolidated and reviewed with consideration for the intended context of use and in light of the other supporting evidence. Following Phase I, cognitive interviews (Phase II) were used to assess participants’ overall understanding of the new PRO instrument (Table 1).

**Table 1:** Details of the interviewers, procedures, and analysis of the qualitative interviews

	Phase I: Concept elicitation interviews	Phase II: Cognitive interviews
Interview description	Concept elicitation focus group discussions and oneonone interviews consisted of a series of openended questions that were designed to facilitate a general discussion on the symptom concepts that are important for patients with EPI, their impacts, and their treatment experience	Cognitive interviews were conducted to assess the clarity, ease of completing, and appropriateness of the format and response scales used in the EPI-SQ, a 12item^a^ (0–4 scale) instrument, used to rate severity of symptoms in the past 7 days in adult patients with EPI due to CP or pancreatectomy.
Interviewers and/or moderators	Health outcomes research professionals trained and experienced in qualitative data collection techniques	
	Interviewers (AS, KC, RS, SM) were female, CL was male)	
	Moderator (SM) and co-moderator (KC) were female	
	Interviewers and/or moderators were employees of Evidera, a research consulting organization, contracted for the study	
	Interviewers and participants had no exposure to or knowledge of each other prior to study commencement	
Interview procedures	Clinical site personnel scheduled focus group discussions or one-on-one interviews (with the help, as needed, from an Evidera staff member) for eligible participants	
	Participants attended either a focus group discussion or a one-on-one interview, which was audio-recorded, with the participants’ permission. Focus groups with 3–5 participants were conducted in person at the clinical sites and lasted approximately 90–120 min. One-on-one interviews were conducted in person or via telephone and lasted approximately 60–90 min. Focus group co-moderators and interviewers took field notes during data collection. When focus groups were supplemented or replaced by one-on-one interviews to expedite recruitment, the discussion followed the same outline, and the semistructured discussion guide was adapted by the interviewer to be suitable for a one-on-one discussion rather than a group discussion	Participants attended one-on-one cognitive interviews on-site or via telephone that lasted approximately 60–90 min and were audio-recorded, with the participants’ permission. Interviewers used a semi-structured qualitative interview guide to conduct cognitive interviews and took field notes.
	Interview audio files were transcribed word-for-word by a professional transcriptionist, which were reviewed by the Evidera staff (KC, RS, CJL, AS) for accuracy and to remove any identifiable personal information	
	Transcripts were not returned to participants	
Analysis	Evidera coding team (KC, RS, SM, CJL) was involved in coding of the transcripts	
	A preliminary coding dictionary was developed for the analysis of the concept elicitation data. The transcripts were reviewed, and words and phrases provided by the participants were identified and coded (per the coding dictionary) as they appeared in the data. A team discussion and revision of the coding scheme refined the concepts and respective definitions.	During analysis, the qualitative data collected as part of this phase of the study were used to evaluate: (1) the content coverage of the new scales to ensure items fully capture all important symptoms of EPI experiences; (2) the clarity of the items within the scale; (3) how the participants interpret the items; (4) ease of completion of the scale; (5) comprehensiveness and relevance of the measure; and (6) the appropriateness of the format, response scales, and recall period.

*CP* chronic pancreatitis, *EPI* exocrine pancreatic insufficiency, *EPI-SQ* EPI Symptom Questionnaire

^a^A 14-item version of the EPI-SQ was field tested during cognitive interviews. Two items were removed based on feedback from cognitive interviews, creating the 12-item version of the EPI-SQ

### Participants

Participants were recruited through five community and academic-based clinical sites within the United States (Louisiana, Maryland [Baltimore, only for Phase I, and Chevy Chase, only for Phase II], Illinois [only for Phase I], and New York [only for Phase II]) using convenience sampling. The study was approved by a central institutional review board (IRB) (Ethical and Independent Review Services) and local IRB approval was received for Phase I (Mercy Medical Center review board: MMC 2014-30 and University of Chicago review board: IRB14-0773) and for Phase II interviews (University of Rochester Medical Center review board: RSRB00056232). All participants provided written informed consent to participate and permission for the interviews to be audio-recorded and transcribed.

The site investigators determined the eligibility of participants by reviewing their medical databases, charts, and appointment schedules to identify participants with EPI who met the pre-specified study eligibility criteria. Clinical site staff used IRB-approved screening scripts to present the study to prospective participants in a consistent manner over the phone or in person. Inclusion/exclusion forms were used by clinical site staff to document participant eligibility. The initial study eligibility criteria were reviewed by the research team, and the original requirement to enroll two subgroups based on most recent fecal pancreatic elastase value of (1) < 128 mcg/g and (2) 128 to 200 mcg/g was relaxed during Phase I to address enrollment challenges and facilitate recruitment of the sample size.

The final inclusion criteria for both Phase I and Phase II interviews required participants to be English-speaking adults (≥ 18 years), diagnosed with EPI (most recent fecal pancreatic elastase value ≤ 200 mcg/g) and CP or evidence of CP (based on computed tomography scan, endoscopic ultrasound, magnetic resonance imaging, or magnetic resonance cholangiopancreatography), or with history of total pancreatectomy or extensive pancreatic resection leading to steatorrhea and resolved with PERT. Current or past users of PERT within 3 months prior to screening were also included.

Participants were excluded if they were diagnosed with CF, celiac disease, Crohn’s disease, ileus or acute abdomen, or any type of malignancy involving the digestive tract, including pancreatic cancer within 5 years of screening. Any participant with clinically relevant medical or psychiatric conditions that, in the judgment of the site investigator, would interfere with completing the study procedures was also excluded.

### Interview procedures: concept elicitation

Enrolled participants attended either a focus group discussion or a one-on-one interview at a time that was convenient for them (Table 1). Focus groups with 4–8 participants were conducted in person at the clinical sites and lasted approximately 90–120 min. One-on-one interviews were conducted in person or via telephone and lasted approximately 60–90 min. Concept elicitation included open-ended questions to elicit participants’ experience and impact of EPI.

Interviewers were trained in qualitative interview techniques and used a semi-structured qualitative interview guide (Supplementary Table S1). Focus group discussions were led by an experienced moderator and assisted by a co-moderator (Table 1).

Participants were asked about the symptoms that they experienced as a result of having EPI, how they would describe their symptom(s), how their symptom(s) vary or change (i.e., frequency, duration, severity), and which EPI symptoms are most important or least important to their experience. Participants were also asked to indicate how their EPI symptoms and treatment had impacted their life.

Following their participation in the qualitative discussions, participants completed questionnaires on socio-demographics and the EPI symptoms. The Evaluation Tool for Rating Presence of EPI Before and After PERT is an 8-item questionnaire that was modified for this study to ask participants to rate the EPI symptoms over the past 7 days [14]. The questionnaire consists of eight items that are rated on a 5-point scale (1 = never, 5 = always). Clinical site personnel also completed a clinical data form for each participant.

### Instrument development

Development of the EPI-SQ followed the recommendations for establishing and reporting evidence in newly developed PRO instruments as found in the ISPOR PRO good research practices for content validity [9, 10]. Participants’ descriptions of EPI symptoms from concept elicitation interviews (Phase I), along with literature and input from clinical experts, were used to develop the EPI-SQ, the new PRO instrument. Feedback from clinical experts was iterative. Initial clinical expert feedback (during Phase I) included a brief discussion of their professional background, including experience of treating patients with EPI, diagnosis and treatment of EPI, the severity of EPI symptoms and their impacts on patients’ lives, and discussion of PRO measures.

An initial pool of items was used to select and refine items based on their relevance and endorsement of concepts from participants during concept elicitation interviews (Phase I). Appropriateness of having a severity or frequency scale was determined based on the participants’ quotes from Phase I interviews. Instructions, questions, recall period, and response scale for the new instrument were based on best practices outlined for the development of PRO instruments and their clinical relevance [8–10]. Once the EPI-SQ was developed, clinical experts were again consulted for feedback on the newly developed questionnaire. Feedback was also sought from Evidera’s internal Cultural & Linguistic Validation Committee team during the development of the questionnaire to consider optimal wording selection should the measure undergo translation in the future.

### Draft EPI-SQ

A draft EPI-SQ was developed, comprising 14 items representing the most salient and seminal symptoms of EPI based on the findings of the concept elicitation work. It consisted of the following items: abdominal pain, bloating, nausea, vomiting, gas [flatulence], constipation, foul smelling stool, greasy or fatty stools, abdominal cramps, diarrhea, lack of energy, lack of appetite, tiredness, and digestion problems with a recall period of “past 7 days” and responses for each item measured on a 0–4 scale (None [0], Mild [1], Moderate [2], Severe [3], and Very Severe [4]).

### Phase II: cognitive interviews

Participants who completed concept elicitation interviews (Phase I) were allowed to participate in cognitive interviews (Phase II). Eligible participants attended one-on-one cognitive interviews on-site or via telephone that lasted approximately 60–90 min and were audio-recorded, with the participants’ permission. Interviewers used a semi-structured qualitative interview guide (Table 1) to conduct cognitive interviews.

Participants completed the EPI-SQ and were then verbally probed on the content of the instrument (i.e., relevance and comprehensiveness, understanding of the instructions, items, response scale, and recall period; Supplementary Table S1).

### Assessment of socio-demographics, EPI tool, and clinical characteristics

After the interview, participants completed a socio-demographic questionnaire and the EPI tool for the purposes of sample description. Clinical site personnel completed a clinical data form for each participant.

The socio-demographic questionnaire included questions on the following characteristics: age, sex, and race/ethnicity, marital status, current living/domestic situation, employment status, educational status, and participants’ EPI symptom experience (severity and impacts).

The Evaluation Tool for Rating Presence of EPI Before and After PERT [14] was used to rate current EPI symptoms for purposes of sample description as was done in the concept elicitation interviews phase.

During concept elicitation interviews (Phase I), details regarding participant’s clinical diagnosis, disease duration, and EPI treatment were captured in the clinical data form. During cognitive interviews (Phase II), the clinical data form also captured three additional details about any condition linked to the participant’s EPI diagnosis, history of taking opioids, and any comorbid conditions.

### Analysis

Qualitative data were analyzed by content analysis [15, 16]. Audio-recorded interviews from Phase I and Phase II were transcribed into written form. Separate coding dictionaries were developed for concept elicitation interviews (Phase I) and cognitive interviews (Phase II). Coding dictionaries were developed to align with the objectives of each phase (concept elicitation or cognitive testing) and were focused on identifying and understanding the concepts of interest or on questionnaire feedback. The coding scheme was discussed by the team, and the concepts and respective definitions were refined. Each transcript was then coded using these respective coding dictionaries by one team member and then reviewed by a second team member; any issues or coding questions were resolved by discussion with the study team.

For both Phase I and Phase II, transcripts were coded using ATLAS.ti (version 7.5.12 or higher; ATLAS.ti Scientific Software Development GmbH, Berlin), a qualitative data analysis software, to conduct a systematic assessment of participants’ feedback [17].

The research team systematically identified and documented themes related to the participants’ experiences with EPI in the Phase I data. Research team members, including those who conducted the focus groups or concept elicitation interviews, and clinical experts, evaluated the data and identified major themes. Saturation was considered to be achieved when no substantially new concepts were captured by including additional study participants [18]. Cognitive interview data (Phase II) were used to evaluate EPI-SQ content validity by assessing the participants’ understanding of the questionnaire as a whole, instructions, items, response scale, and recall period of the EPI-SQ (more details in Table 1). After Phase II, research team members, including those who conducted the cognitive interviews, met to evaluate the results and consider final modifications to the EPI-SQ.

Quantitative data were analyzed using Microsoft Access and DataFax, an optical character recognition software that is compliant with the US Food and Drug Administration (US FDA) Title 21 Code of Federal Regulations Part 11 compliant (DF/Net Research, Inc., Seattle, WA). Descriptive statistics (mean, standard deviation [SD], frequency) were calculated using SAS version 9.4 (SAS Institute Inc., Cary, NC).

Further details of the interviewers, interview procedures, and the analysis of the qualitative interviews are provided in Table 1.

## Results

### Participants and socio-demographic characteristics

Twenty-one participants completed concept elicitation interviews (Phase I) (Table 2); 61.9% were female, 90.5% were non-Hispanic/Latino,, 52.4% were white, and the mean (SD) age was 55.4 (13.1) years. Self-reported severity of EPI symptoms was moderate in nine (42.9%), severe in four (19.0%), and mild in three participants (14.3%). Five participants (23.8%) self-reported having no EPI symptoms.

**Table 2:** Concept elicitation and cognitive interviews: Socio-demographic characteristics (self-reported)

Characteristics	Phase I: concept elicitation interviews (*N* = 21)	Phase II: cognitive interviews (*N* = 7)
Age (years), mean (SD)	55.4 (13.1)	61.9 (11.8)
Sex, n (%)		
Male	8 (38.1)	3 (42.9)
Female	13 (61.9)	4 (57.1)
Ethnicity, n (%)		
Hispanic or Latino	1 (4.8)	0 (0.0)
Non-Hispanic/Latino	19 (90.5)	6 (85.7)
Missing	1 (4.8)	1 (14.3)
Race, n (%)		
Asian	1 (4.8)	0 (0.0)
Black or African American	6 (28.6)	3 (42.9)
White	11 (52.4)	4 (57.1)
Other^a^	3 (14.3)	0 (0.0)
Marital status, n (%)		
Single	2 (9.5)	2 (28.6)
Married	14 (66.7)	4 (57.1)
Divorced/Separated	4 (19.1)	1 (14.3)
Widowed	1 (4.8)	0 (0.0)
Current living/Domestic situation, n (%)		
Living alone	2 (9.5)	3 (42.9)
Living with a partner or spouse, family, or friends	19 (90.5)	4 (57.1)
Employment status, n (%)		
Full-time work	6 (28.6)	2 (28.6)
Part-time work	3 (14.3)	0 (0.0)
Homemaker/Housewife	1 (4.8)	0 (0.0)
Retired	6 (28.6)	1 (14.3)
Disabled	8 (38.1)	2 (28.6)
Other^b^	0 (0.0)	1 (14.3)
Missing	0 (0.0)	1 (14.3)
Educational status, n (%)		
Less than high school	1 (4.8)	0 (0.0)
High school, GED/High school equivalent	10 (47.7)	0 (0.0)
Some college education	3 (14.3)	1 (14.3)
Graduated 2-year college	2 (9.5)	0 (0.0)
Completed college degree	2 (9.5)	0 (0.0)
Some postgraduate education	1 (4.8)	2 (28.6)
Completed postgraduate degree	1 (4.8)	2 (28.6)
Other^c^	1 (4.8)	1 (14.3)
Missing	0 (0.0)	1 (14.3)
Severity of EPI symptoms, n (%)		
None	5 (23.8)	1 (14.3)
Mild	3 (14.3)	3 (42.9)
Moderate	9 (42.9)	1 (14.3)
Severe	4 (19.0)	0 (0.0)
Missing	0 (0.0)	2 (28.6)
Severity of EPI impacts, n (%)		
None	2 (9.5)	2 (28.6)
Mild	5 (23.8)	1 (14.3)
Moderate	8 (38.1)	1 (14.3)
Severe	6 (28.6)	0 (0.0)
I do not know	0 (0.0)	1 (14.3)
Missing	0 (0.0)	2 (28.6)

*EPI* exocrine pancreatic insufficiency, *GED* general equivalency diploma, *SD* standard deviation

^a^Other: American (*n* = 1); Puerto Rican (*n* = 1)

^b^Self-employed

^c^Received vocational training

Seven participants completed cognitive interviews (Phase II) (Table 2); 57.1% were female, 85.7% were non-Hispanic/Latino, 57.1% were white, and the mean (SD) age was 61.9 (11.8) years. Self-reported severity of EPI symptoms was mild in three participants (42.9%), moderate in one participant (14.3%), and reported as “none” in one participant (14.3%). None of the participants reported EPI symptoms as “severe.” Two participants did not provide a response to this question.

### Clinical characteristics

Among participants who took part in concept elicitation interviews (Phase I), mean (SD) time since original diagnosis was 41.8 (49.5) months, and 57.1% had fecal elastase of ≥ 50 mcg/g to < 200 mcg/g (Table 3). All participants were on EPI medication, with 76.2% receiving CREON^®^ at the time of enrollment (mean [SD] length of treatment of 22.7 [22.6] months). Clinician-reported severity of EPI symptoms was mild in nine (42.9%), none (no symptoms present) in six (28.6%), and moderate or severe in three participants each (14.3%).

**Table 3:** Concept elicitation and cognitive interviews: clinical characteristics (Clinician-reported)

Characteristics	Phase I: concept elicitation interviews (*N* = 21)	Phase II: cognitive interviews (*N* = 7)
Duration in practice (months), mean (SD)	34.3 (39.4)	70.3 (77.2)
Original diagnosis (months), mean (SD)	41.8 (49.5)	35.5 (39.6)
Fecal elastase value (mean calculated based on sub-group with values) (%)		
Not applicable – Total pancreatectomy (0)	2 (9.5)	0 (0.0)
≥ 15 mcg/g and < 50 mcg/g	2 (9.5)	0 (0.0)
< 50 mcg/g	5 (23.8)	2 (28.6)
≥ 50 mcg/g and < 200 mcg/g	12 (57.1)	5 (71.4)
Fecal elastase value (mean calculated based on sub-group with values)^a^, mean (SD)	90.1 (47.9)	91.7 (55.4)
EPI medication at the time of enrollment, n (%)		
No	0 (0.0)	1 (14.3)^b^
Yes	21 (100.0)	6 (85.7)
CREON^®^	16 (76.2)	4 (66.7)
ZenPep	4 (19.0)	2 (33.3)
Pancrease	1 (4.8)	0 (0.0)
Length of treatment regimen (months), mean (SD)	22.7 (22.6)	21.1 (28.7)
Clinical global impression of severity of EPI symptoms, n (%)		
0 = none (symptoms not present)	6 (28.6)	0 (0.0)
1 = mild (symptoms present, but not bothersome)	9 (42.9)	2 (28.6)
2 = moderate (symptoms bothersome)	3 (14.3)	4 (57.1)
3 = severe (symptoms interfere with normal activities)	3 (14.3)	1 (14.3)

*EPI* exocrine pancreatic insufficiency, *SD* standard deviation

^a^Concept elicitation, *n* = 14; cognitive interviews, *n* = 6

^b^Marked as “No” at enrollment; however, the participant while completing the form specified as “Reports starting soon - used CREON^®^ in past”

Among participants who took part in cognitive interviews (Phase II), the mean (SD) time since original diagnosis was 35.5 (39.6) months, and 71.4% had fecal elastase of ≥ 50 mcg/g to < 200 mcg/g (Table 3). Most participants (85.7%) were on EPI medication, with 66.7% receiving CREON^®^ at the time of enrollment (mean [SD] length of treatment of 21.1 [28.7] months). Clinician-reported severity of EPI symptoms was moderate in four (57.1%), mild in two participants (28.6%), and severe in one participant (14.3%).

### EPI tool for rating presence of EPI symptoms

Each of the eight EPI symptoms (i.e., feeling of indigestion, abdominal cramps after meals, large amounts of gas, foul smelling gas or stools, floating or greasy or fatty stools, frequent stools, loose stools, and weight loss) had been experienced by over half the participants (53%) who took part in concept elicitation interviews (Phase I) (Table 4). Abdominal cramps after meals (42.9%), loose stools (42.9%), and foul-smelling gas or stools (38.1%) were most frequently rated as “sometimes.”

**Table 4:** Sample characteristics: rating of presence of EPI symptoms (Patient-reported)

Symptom	Phase I: concept elicitation interviews (*N* = 21)	Phase II: cognitive interviews (*N* = 7)
Feelings of indigestion (%)		
Never	6 (28.6)	1 (14.3)
Rarely	4 (19.0)	2 (28.6)
Sometimes	6 (28.6)	2 (28.6)
Often	4 (19.0)	2 (28.6)
Always	1 (4.8)	0 (0.0)
Abdominal cramps after meals (%)		
Never	4 (19.0)	2 (28.6)
Rarely	5 (23.8)	3 (42.9)
Sometimes	9 (42.9)	1 (14.3)
Often	2 (9.5)	0 (0.0)
Always	1 (4.8)	0 (0.0)
Missing	0 (0.0)	1 (14.3)
Large amounts of gas (%)		
Never	1 (4.8)	1 (14.3)
Rarely	4 (19.0)	3 (42.9)
Sometimes	6 (28.6)	1 (14.3)
Often	6 (28.6)	0 (0.0)
Always	4 (19.0)	1 (14.3)
Missing	0 (0.0)	1 (14.3)
Foul smelling gas or stools (%)		
Never	1 (4.8)	1 (14.3)
Rarely	2 (9.5)	3 (42.9)
Sometimes	8 (38.1)	1 (14.3)
Often	5 (23.8)	2 (28.6)
Always	5 (23.8)	0 (0.0)
Floating or greasy or fatty stools (%)		
Never	8 (38.1)	3 (42.9)
Rarely	1 (4.8)	3 (42.9)
Sometimes	6 (28.6)	1 (14.3)
Often	3 (14.3)	0 (0.0)
Always	1 (4.8)	0 (0.0)
Missing	2 (9.5)	0 (0.0)
Frequent stools (%)		
Never	2 (9.5)	2 (28.6)
Rarely	7 (33.3)	1 (14.3)
Sometimes	6 (28.6)	3 (42.9)
Often	4 (19.0)	1 (14.3)
Always	1 (4.8)	0 (0.0)
Missing	1 (4.8)	0 (0.0)
Loose stools (%)		
Never	4 (19.0)	2 (28.6)
Rarely	4 (19.0)	2 (28.6)
Sometimes	9 (42.9)	2 (28.6)
Often	3 (14.3)	1 (14.3)
Always	0 (0.0)	0 (0.0)
Missing	1 (4.8)	0 (0.0)
Weight loss (%)		
Never	6 (28.6)	2 (28.6)
Rarely	4 (19.0)	3 (42.9)
Sometimes	4 (19.0)	1 (14.3)
Often	4 (19.0)	1 (14.3)
Always	3 (14.3)	0 (0.0)

*EPI* exocrine pancreatic insufficiency

Each of the eight EPI symptoms had been experienced by at least four participants (57%) who took part in cognitive interviews (Phase II) in the previous 7 days (Table 4). Abdominal cramps after meals, large amounts of gas, foul smelling gas or stools, floating or greasy or fatty stools, and weight loss (42.9% each) were most frequently rated as “rarely.”

### Phase I: concept elicitation interviews

#### Symptom experience

Participants reported a variety of EPI symptoms. Symptoms endorsed by participants, along with quotes about their symptom experience, are provided in Table 5. Abdominal pain was the most frequently reported EPI symptom (90% of participants endorsed this concept). Pain was commonly discussed in terms of severity, but also described in terms of frequency and duration. Descriptions of abdominal pain included “unbearable” and “severe” pain. Nausea or vomiting was endorsed by 76% participants, with mentions of vomiting twice a week, “a lot” of nausea, and dry heaving. Over two-thirds of participants (71%) endorsed abdominal bloating, relating it to swelling of their stomach, and describing it as “sharp, tight” and “severity of 8 on a scale of 1–10.” Constipation was endorsed by over two-thirds of participants (71%), with mentions of discomfort, bowel movements that “sits there” and “don’t move” in the digestive system, and use of suppositories. Appetite changes (i.e., reduced appetite or eating reduced amounts) were experienced by 67% of participants, who felt “packed full” even after a couple of bites. Change in energy, gas, or flatulence, eating and digestion or bowel movements, and weight loss were each endorsed by 62% of participants.

**Table 5:** Representative quotes for the symptom concepts

Symptom concepts^a^	Participants reporting, *n* (%)	Representative quotes (Participant ID)
Abdominal pain	19 (90%)	The pain, the pains are very unbearable… It’s severe pain, oh, God, it looked like it just grabbed you in the stomach and it wouldn’t let go, yes (002–011, 1 year since EPI diagnosis, EPI severity none – symptoms not present)
		Yeah, I can feel my pain, it’s like on and off, it’s something—it’s really worse,… (002–009, 8 years since EPI diagnosis, mild EPI severity)
Nausea or vomiting	16 (76%)	Well, I mean nausea is nausea,…give me something for pain, and give me something to throw up in. And it was just dry heaving (001–001, 6 years since EPI diagnosis, mild EPI severity)
Abdominal bloating	15 (71%)	… my whole stomach was swollen—the packed feeling was at the top (001–001, 6 years since EPI diagnosis, mild EPI severity)
		Sharp, tight. At least an 8 on a scale of 1 to 10 (002 − 001, 6 years since EPI diagnosis, mild EPI severity)
Constipation	15 (71%)	…I mean once the — let’s just say it go to the point where if I didn’t do a suppository, I wasn’t going (003–021, 1 year since EPI diagnosis, EPI severity none – symptoms not present)
		It sits there and don’t move.( )— little uncomfortable. (…). It sits there and it’s like when it moves I’m like oh, finally…(003–019, 1 year since EPI diagnosis, severe EPI severity)
Appetite changes	14 (67%)	I didn’t have an appetite…anything would make me just feel packed full, um, just one or two bites, and I just couldn’t eat anymore, I didn’t have an appetite (001–001, 6 years since EPI diagnosis, mild EPI severity)
Change in energy	13 (62%)	Oh, yeah. I don’t have no energy (001–003, 1 year since EPI diagnosis, mild EPI severity)
		Oh, my God, yes, I don’t have very much energy at all unless I have a good day and everything’s working right […]I might get two days out of a month that I might feel pretty good (002 − 001, 6 years since EPI diagnosis, mild EPI severity)
Gas or flatulence	13 (62%)	…I’d still have a lot of gas and it’s a smelly gas…it’s the gas problem…(001–003, 1 year since EPI diagnosis, mild EPI severity)
		Well, it was real frequent before [treatment], but now it just comes every now and then (002–011, 1 year since EPI diagnosis, EPI severity none – symptoms not present)
Eating and Digestion or bowel movements	13 (62%)	Well, I don’t eat, uh, a lot of oily foods…like you have hard fried food, I can’t eat that (002–011, 1 year since EPI diagnosis, EPI severity none – symptoms not present)
		…I don’t eat fried foods (002–009 [8 years since EPI diagnosis, mild EPI severity] and 002–011 [1 year since EPI diagnosis, EPI severity none – symptoms not present])
Weight loss	13 (62%)	…I had weight loss,…but I wasn’t really aware of that until the doctor just recently pointed out that I’d lost five pounds…(002–012, 4 years since EPI diagnosis, EPI severity none – symptoms not present)
		I’m losing weight but I know the problem probably is that because I don’t have an appetite (001–003, 1 year since EPI diagnosis, mild EPI severity)
Indigestion	12 (57%)	Um, yeah, historically I would have indigestion with [EPI] (003–011, 19 years since EPI diagnosis, moderate EPI severity)
Cramping	12 (57%)	…it was absolutely so painful that I would just like cry and try to roll myself over just to get going. That’s how bad the cramping… (003–004, 1 year since EPI diagnosis, severe EPI severity)
Urgent bowel movements	10 (48%)	Urgency, but not accidents [bowel movements] (002–011 and 002–010 [1 year since EPI diagnosis, mild EPI severity])
Foul smelling stools	9 (43%)	…it was awful, it smelled—well, honestly, it smelled like it had soured inside of me…(001–001, 6 years since EPI diagnosis, mild EPI severity)
		Pretty rotten. Uh, it’s any wonder my guts hurt the way it smells (002 − 001, 6 years since EPI diagnosis, mild EPI severity)
Diarrhea	9 (43%)	Every two hours and then every other day… (002–014, 3 years since EPI diagnosis, severe EPI severity)
Change in stool consistency	9 (43%)	Before [medication], like, as I said, constipation, then diarrhea out of nowhere (002–010 [1 year since EPI diagnosis, mild EPI severity])
		…today it might be really nice and firm, and then tomorrow probably something—it was just up and down, but now since I take the medicine I don’t have the problem (002–010 [1 year since EPI diagnosis, mild EPI severity] and 002–011 [1 year since EPI diagnosis, EPI severity none – symptoms not present])
Greasy or fatty or floating stools	9 (43%)	Um, I’ve seen it so I will yes, I’ve seen it greasy or look fattening (001–003)
		…Sometimes it floats (001–002, 1 year since EPI diagnosis, moderate EPI severity)

*EPI* exocrine pancreatic insufficiency

^a^Other symptom concepts endorsed by participants include: difficulty flushing toilet because of the stool consistency, muscle loss, and distention (*n* = 3, 14% each); heartburn, frequent bowel movements, and difficulty swallowing (*n* = 2, 10% each); bloody stool, bruises, chest pain, difficulty digesting food, dizziness, dry cough, headache, numbness and salty tongue, rusty urine, shortness of breath, spasms, sweating, and uncomfortable (*n* = 1, 5% each)

#### Impact of living with EPI

Focus group participants discussed ways in which their lives were impacted by EPI. Several impacts were endorsed by participants. Lifestyle changes were the most frequently mentioned impact (86%), followed by effects on social activities (43%).

##### Saturation of symptom and impact concepts

Eighteen symptom concepts were endorsed by the first group of interviewed participants (*n* = 5). Two new symptom concepts (heart burn and frequent bowel movements) were endorsed in the second group (*n* = 3) and difficulty swallowing, and distention were the new symptom concepts endorsed in third and fourth groups (*n* = 4 each), respectively (Supplementary Table S2). Nine impact concepts were endorsed by the first group of interviewed participants (*n* = 5), whereas only one new impact (other impacts) emerged from the third group (*n* = 3). No new concepts emerged in the fourth group (*n* = 4) (for impacts) or fifth group (*n* = 5) (for symptoms), indicating that saturation was achieved (i.e., no additional interviews were required).

### Instrument development and refinement

The EPI-SQ was developed primarily using the data from concept elicitation interviews (Phase I), which were further supplemented by literature and input from clinical experts. Items were written to represent the participants’ experience. Language used by participants to describe the important aspects of their symptom experience was considered when choosing appropriate wording of the items. Supporting quotes from participants were used to evaluate the inclusion or exclusion of certain items. Based on the review of how participants defined their symptoms from concept elicitation (Phase I) interviews, a severity scale was identified as the best response scale for the EPI-SQ.

Once the EPI-SQ was developed, four clinical experts were consulted for feedback on the questionnaire, and additional probing was added to the cognitive interview guide to address some comments, but no changes were made to the EPI-SQ after consultation with clinical experts.

### Phase II: cognitive interviews

All seven participants (100%) completed the EPI-SQ and provided their first impressions of the questionnaire as follows:

- **Relevance**: Six (86%) indicated that the questionnaire was relevant to their symptom experience, clear, and easy to understand. One (14%) participant indicated that the questionnaire was repetitive.
- **Recall period**: Four (67%) of the six participants who were asked to describe their understanding of the recall period correctly interpreted the past 7 days to mean the “last week.” One participant (14%) felt a 7 day timeframe would not accurately capture their EPI symptom experience, as their EPI symptoms does not always show up in 7 days. However, upon further probing, the participant indicated that completing the questionnaire every week for at least 2–3 months would capture variability in their symptoms better.
- **Instructions**: Instructions on the EPI-SQ were well understood by all seven participants (100%). When participants were given alternative instructions with recall period changed to “past 24 hours,” four (57%) preferred the original instructions (i.e., recall period of “past 7 days”), two (29%) indicated no preference (because their symptoms were constantly the same), and one participant (14%) did not express a clear preference between the two options given. Most participants (86%) reported no difficulty with the response options and indicated they were understood as intended.

#### Understanding of symptom items and suggestions

During discussion, participants were asked about their understanding of the EPI symptom items and any suggestions for changes to the items.

- **Understanding of items and response options**: All participants demonstrated clear understanding of all symptom items as intended, except digestion problems. One participant (14%) indicated the phrase “digestion problems” was confusing and suggested to change it to “heartburn” or “indigestion.” When participants compared the terms “lack of energy” and “tiredness”, three (43%) indicated that they are two different concepts. Additionally, when reviewing the “tiredness” item, one participant (14%) suggested adding a parenthesis next to the term “tiredness” and include “lack of energy”. When asked to provide their response, participants mostly selected the response option of “none” or “mild” for the majority of items.
- **Comprehensiveness**: When asked about any missing symptoms, the majority of participants (86%) did not have suggestions around missing items. One participant (14%) indicated that back pain and hospitalizations due to pain were missing.
- **Alternate wording**: Participants were also provided the option to choose between the original or an alternate wording for each of the 14 items: starting the item with a phrase “During the past 7 days” and presenting the items as questions to describe “how bad” their symptom was, instead of statements to “rate” them. The majority of participants preferred the original wording over the alternate wording.

### Final modifications to the EPI-SQ

Two items were removed from the EPI-SQ following feedback from participants in cognitive interviews (Phase II) or Evidera’s internal Cultural & Linguistic Validation Committee team (“Rate your digestion problems during the past 7 days” and “Rate your lack of energy during the past 7 days”). The item, “Rate your digestion problems during the past 7 days,” was deleted due to inconsistent interpretation by the cognitive interview participants. The item, “Rate your lack of energy during the past 7 days,” was deleted based on feedback from participants that this item represented a similar concept to “tiredness” (“Rate your tiredness during the past 7 days”). Feedback from Evidera’s internal Cultural & Linguistic Validation Committee team also indicated that “tiredness” may be easier to translate in future versions, and that it could be difficult to differentiate between “lack of energy” and “tiredness” in some languages. The final version of the questionnaire has 12 items. In all cases where alternate wording options were tested, the original wording was retained in the final version of the questionnaire.

## Discussion

To the best of our knowledge, this is the first qualitative study to develop and assess an EPI-specific PRO questionnaire in the US patient population. Findings demonstrate that the content of the EPI-SQ is relevant to patients with EPI and comprehensively covers concepts about their EPI symptom experience. In concept elicitation interviews (Phase I), among EPI symptom concepts, abdominal pain was reported by most participants. They also indicated that EPI symptoms had an impact on their lifestyle. Most symptoms and impacts emerged in the first group of interviews and saturation was achieved by the fourth or fifth group of interviews.

The EPI-SQ was developed based on the best practices recommended for development and validation of PRO instruments [8–10]. The questionnaire includes typical symptoms of EPI [4], thus offering more specificity over instruments that are generic (e.g., SF-36) [11] or those that measure overall gastrointestinal experience (e.g., GIQLI) [13]. Since the GIQLI was designed to assess specific health-related quality of life and gastrointestinal symptoms in patients with various gastrointestinal diseases, it may not be able to capture detailed and specific symptom aspects of EPI when assessing patients diagnosed with EPI. Moreover, the GIQLI captures frequency of symptoms rather than severity, measures both impacts and symptoms on a single scale, and includes 36 items [13], which could be burdensome for patients to complete in real-world clinical practice. While the PEI-Q and EPI-SQ have item overlap, the tools were developed in different populations. Although the PEI-Q was validated in patients with CP and CF, half of the patient population was enrolled in the study solely based on the diagnosis of their EPI by clinical experts, as information about diagnostic tests was unavailable.

A key difference between the PEI-Q and the EPI-SQ is that the PEI-Q includes both symptoms and impacts in a total summary score, making interpretation difficult. The EPI-SQ focuses on symptoms which are more proximal to the patient experience. If an instrument is being used as a primary or key secondary endpoint in a pivotal clinical trial, regulatory agencies have demonstrated a clear preference for items that are proximal to patients’ disease and treatment experience. Since both symptoms and their impacts are interlinked in conditions like EPI, in order to reduce patient burden as well as to make the EPI-SQ relatively brief, we chose to keep the questionnaire a symptom-focused tool [3, 19, 20]. The EPI-SQ with fewer items (12 items in the final version of the questionnaire) is less burdensome for patients to complete, thus making it more suitable in a clinical trial or real-world clinical practice.

Two items each on lack of energy and digestion problems were removed from the final version of the EPI-SQ based on the feedback during cognitive interviews (Phase II) or Evidera’s internal Cultural & Linguistic Validation Committee team. The EPI-SQ fulfils the need for a content-valid, EPI-specific instrument for assessing symptom severity and their impacts in the US patient population diagnosed with EPI. Results of cognitive interviews (Phase II) suggested that items on the EPI-SQ were relevant to patients with EPI and that they understood the items, instructions, and response options. Most of the participants felt that none of the key symptoms of EPI were missing from the EPI-SQ. Although one participant indicated that back pain and hospitalizations due to pain were missing, influence of such factors can also be verified from other secondary sources, such as patient’s electronic medical records.

Recruitment difficulties and recruitment of participants from a single country limit generalizability of the present study findings to the overall population of patients diagnosed with EPI encountered in real-world clinical practice. The cognitive interview (Phase II) sample size was lower than initially targeted due to continued recruitment challenges in this population. However, the study was designed and carried out in accordance with accepted instrument development standards, including testing in patients representative of the target population and the use of an iterative qualitative research process to confirm content validity [10]. In addition, although small, the sample size was comparable with published suggestions of 5–15 participants per cognitive interview for qualitative research studies of this type. Furthermore, saturation in the concept elicitation phase of the study (Phase I) supports both the validity of the findings and their relevance to patients with EPI, [21] and given the consistency of the overall feedback received on the EPI-SQ, it is unlikely that a larger sample size would have changed the outcomes of the research.

Additionally, some characteristics differed between the Phase I and Phase II samples (e.g., a greater proportion of the Phase II sample had a higher education level [majority some postgraduate or above versus high school or less], lower symptom severity [majority none to mild versus moderate to severe], and shorter mean time since diagnosis [3 years versus 3.5 years] compared with the Phase I sample). Recruitment of patients with CP or pancreatectomy only and excluding patients diagnosed with other underlying pancreatic disorders, such as CF and pancreatic cancer, could be another potential limitation from representativeness perspective. Although EPI symptoms and severity differ widely from patient to patient, some of the most common EPI symptoms (e.g., abdominal pain, bloating, constipation, steatorrhea, weight loss) are presented consistently across other gastrointestinal conditions [4, 22, 23]. Despite being developed in differently diagnosed populations from different countries, both the PEI-Q and EPI-SQ have conceptual overlap of symptom items, thus non-inclusion of patients with other underlying pancreatic disorders may not have a significant influence on the outcomes. Finally, although the EPI-SQ was primarily developed to monitor EPI symptoms in clinical practice, the recall period of “past 7 days” could be a limitation for its use in clinical trials. The US FDA guidance for other gastrointestinal conditions recommends a recall period of “past 24 days” to capture their symptoms [24].

Under-diagnosis and under-treatment are significant issues in the EPI patient population, which are also consequently correlated with aggravated symptom experience and poor treatment adherence, respectively, leading to sub-optimal therapeutic outcomes. This study evaluated symptom experience in patients with EPI and assessed the content validity of the EPI-SQ. Hence, the EPI-SQ can be used as a standardized tool in routine clinical practice to monitor disease status as well as treatment effectiveness through symptom assessment, a key parameter in EPI management.

## Conclusion

The qualitative evidence collected in this study support the content validity of the EPI-SQ in measuring EPI symptom severity in US patient populations diagnosed with EPI. The new, EPI-specific PRO instrument was clear and well understood by patients diagnosed with EPI. Further research is suggested to establish the psychometric performance and score interpretability of the EPI-SQ.

### Electronic supplementary material

Below is the link to the electronic supplementary material.

Supplementary Material 1

## Data Availability

All data generated or analyzed during this study are included in this article [and its supplementary information file].

## Abbreviations

CP: Chronic pancreatitis
CF: Cystic fibrosis
EORTC QLQ-C30: European Organisation for Research and Treatment of Cancer Quality of Life Questionnaire C-30
EPI: Exocrine pancreatic insufficiency
EPI-SQ: Exocrine Pancreatic Insufficiency Symptom Questionnaire
GIQLI: Gastrointestinal Quality of Life Index
IRB: Institutional review board
PAN26 and PAN28: EORTC pancreatic cancer modules
PEI: Pancreatic exocrine insufficiency
PEI-Q: Pancreatic exocrine insufficiency Questionnaire
PERT: Pancreatic-enzyme replacement therapy
PRO: Patient-reported outcome
SF-36: 36-item Short Form of the Medical Outcomes Study
US FDA: US Food and Drug Administration
