# Supplementary material for: Developing the EPI Symptom Questionnaire (EPI-SQ): a qualitative study to understand the symptom experience of patients with exocrine pancreatic insufficiency (EPI)
Source: J Patient Rep Outcomes. 2024 Jul 25;8:80. doi: 10.1186/s41687-024-00760-6 (PMC11282023; doi:10.1186/s41687-024-00760-6)
Supplement: Supplementary file 1 — Supplementary Material 1 [file 41687_2024_760_MOESM1_ESM.pdf]

Supplementary data

Table S1 Outline of the concept elicitation guide.

**Phase I (concept elicitation)**	
**Themes to be explored**	**Examples of questions**
1. History of EPI	Time since diagnosis of EPI?<br>What led to diagnosis?<br>Type of physician diagnosing?
2. EPI symptoms	Participants were probed on their symptom experience; the endorsed symptoms were probed on:<br>• Time since symptom onset?<br>• Time from symptom onset to EPI diagnosis?<br>Description:<br>• Please describe you experience of <<symptom>><br>• Location (if applicable)?<br>• Frequency?<br>• Severity?

	• Timing (time of day, proximity to ingestion)?<br>• Related to ingestion of …?<br>• Consistency (continuous, variable)?<br>Variation:<br>• How does your experience of <<symptom>> vary or change?
3. Treatments for EPI	• What treatments have you tried?<br>• How many EPI treatments have you tried?
4. Impact of EPI symptoms on participant’s life	EPI symptom impact/impact on functioning on participant’s life:<br>• How is your life affected by EPI?<br>Daily activities:<br>• How is your everyday activity affected by EPI?<br>Social activities:<br>• How are your social activities affected by EPI?<br>Relationships:<br>• How are your relationships affected by EPI?

	Work/school:<br>• How is work or school affected by EPI?<br>Other activities:<br>• How are your other activities affected by EPI?
**Phase II (Cognitive Interviews)**	
**Topics**	**Examples of questions**
Feedback on the EPI-SQ	Overall:<br>• What were your first impressions of the questionnaire?<br>• Does it cover the areas important to your experiences?<br>Understanding of the instructions:<br>• In your own words, please tell me what the instructions were asking you to do.<br>• Please describe any confusion or difficulty you had in understanding the instructions.<br>• Please tell me about any words or phrases you would change to improve the instructions.

Understanding of the items and testing of alternate item wording:

- What does <<item>> mean to you?
- How does <<item>> relate to your experience with EPI?
- Rate your <<item>> during the past 7 days

Alternate wording:

During the past 7 days, how bad was your <<item>>?

Understanding of the response scales:

- What did you think of the response options overall?
- Were you able to select a response for each item?
- What caused you to choose <<response>>?
- Please tell me about a time when you might have chosen <<response above / response below>>

Understanding of the recall period:

- When you answered the questions, what period of time did you think about (e.g., today, yesterday, last week, “the past 7 days”)?

EPI, Exocrine pancreatic insufficiency; EPI-SQ: Exocrine Pancreatic Insufficiency Symptom Questionnaire.

Table S2 Saturation grid of symptom concepts.

Symptom concepts*	Group 1† (n = 5)	Group 2‡ (n = 3)	Group 3† (n = 4)	Group 4‡ (n = 4)	Group 5‡ (n = 5)	Total (%)
Abdominal pain	X (5)	X (2)	X (4)	X (3)	X (5)	19 (90)
Nausea or vomiting	X (4)	X (2)	X (4)	X (4)	X (3)	16 (76)
Abdominal bloating	X (4)	X (2)	X (3)	X (3)	X (3)	15 (71)
Constipation	X (5)	X (1)	X (3)	X (2)	X (3)	15 (71)
Appetite changes	X (5)	–	X (2)	X (3)	X (4)	14 (67)
Change in energy	X (5)	–	X (3)	X (2)	X (4)	13 (62)
Gas or flatulence	X (3)	X (2)	X (4)	X (3)	X (1)	13 (62)
Eating and Digestion or bowel movements	X (3)	X (3)	X (3)	X (3)	X (1)	13 (62)
Weight loss	X (4)	–	X (4)	X (3)	X (2)	13 (62)
Indigestion	X (2)	X (2)	X (4)	X (2)	X (2)	12 (57)
Cramping	X (3)	–	X (4)	X (4)	X (1)	12 (57)
Urgent bowel movements	X (1)	X (1)	X (3)	X (3)	X (2)	10 (48)

Foul smelling stools	X (3)	–	X (4)	X (2)	X (1)	9 (43)
Change in stool consistency	X (2)	X (3)	X (1)	X (3)	X (1)	9 (43)
Diarrhea	X (1)	X (3)	X (2)	X (2)	X (1)	9 (43)
Greasy or fatty or floating stools	X (1)	–	X (3)	X (2)	X (3)	9 (43)
Difficulty flushing toilet	X (1)	–	X (1)		X (1)	3 (14)
Muscle loss	X (1)	–	–	X (1)	X (1)	3 (14)
Distention	–	–	–	X (3)	–	3 (14)
Heartburn	–	X (2)	–	–	–	2 (10)
Frequent bowel movements	–	X (1)	X (1)	–	–	2 (10)
Difficulty swallowing	–	–	X (1)	–	X (1)	2 (10)

*Other symptom concepts endorsed by participants (n = 1 each) included: bloody stool, bruises, chest pain, difficulty digesting food, dizziness, dry cough, headache, numbness and salty tongue, rusty urine, shortness of breath, spasms, sweating, and uncomfortable.

†Themes were identified from one-on-one interviews.

‡Themes were identified from focus group discussions.
